# Multiomics in the central Arctic Ocean for benchmarking biodiversity change

**DOI:** 10.1371/journal.pbio.3001835

**Published:** 2022-10-17

**Authors:** Thomas Mock, William Boulton, John-Paul Balmonte, Kevin Barry, Stefan Bertilsson, Jeff Bowman, Moritz Buck, Gunnar Bratbak, Emelia J. Chamberlain, Michael Cunliffe, Jessie Creamean, Oliver Ebenhöh, Sarah Lena Eggers, Allison A. Fong, Jessie Gardner, Rolf Gradinger, Mats A. Granskog, Charlotte Havermans, Thomas Hill, Clara J. M. Hoppe, Kerstin Korte, Aud Larsen, Oliver Müller, Anja Nicolaus, Ellen Oldenburg, Ovidiu Popa, Swantje Rogge, Hendrik Schäfer, Katyanne Shoemaker, Pauline Snoeijs-Leijonmalm, Anders Torstensson, Klaus Valentin, Anna Vader, Kerrie Barry, I.-M. A. Chen, Alicia Clum, Alex Copeland, Chris Daum, Emiley Eloe-Fadrosh, Brian Foster, Bryce Foster, Igor V. Grigoriev, Marcel Huntemann, Natalia Ivanova, Alan Kuo, Nikos C. Kyrpides, Supratim Mukherjee, Krishnaveni Palaniappan, T. B. K. Reddy, Asaf Salamov, Simon Roux, Neha Varghese, Tanja Woyke, Dongying Wu, Richard M. Leggett, Vincent Moulton, Katja Metfies

**Affiliations:** 1 School of Environmental Sciences, University of East Anglia, Norwich, United Kingdom; 2 School of Computing Sciences, University of East Anglia, Norwich, United Kingdom; 3 Stockholm University, Department of Ecology, Environment and Plant Sciences, Stockholm, Sweden; 4 Swedish University of Agricultural Sciences, Department of Aquatic Sciences and Assessment, Uppsala, Sweden; 5 Department of Atmospheric Science, Colorado State University, Fort Collins, Colorado, United States of America; 6 Scripps Institution of Oceanography, University of California San Diego, La Jolla, California, United States of America; 7 Department of Biological Sciences, University of Bergen, Bergen, Norway; 8 Marine Biological Association, Plymouth, United Kingdom; 9 Institute of Quantitative and Theoretical Biology, Heinrich-Heine University Düsseldorf, Düsseldorf, Germany; 10 Alfred-Wegener Institute Helmholtz Centre for Polar and Marine Research, Bremerhaven, Germany; 11 Department of Arctic and Marine Biology, UiT-The Arctic University of Norway, Tromso, Norway; 12 Norwegian Polar Institute, Fram Centre, Tromsø, Norway; 13 NORCE Climate and Environment NORCE, Norwegian Research Centre, Bergen, Norway; 14 School of Life Sciences, University of Warwick, Coventry, United Kingdom; 15 University of Rhode Island, Graduate School of Oceanography, Narragansett, Rhode Island, United States of America; 16 University Centre in Svalbard (UNIS), Forskningsparken, Longyearbyen, Norway; 17 DOE Joint Genome Institute, Lawrence Berkeley National Laboratory, Berkeley, California, United States of America; 18 Earlham Institute, Norwich Research Park, Norwich, United Kingdom; 19 Helmholtz Institute for Functional Marine Biodiversity at the University of Oldenburg, Oldenburg, Germany; Smithsonian Institution, UNITED STATES

## Abstract

The MOSAiC ice-drift study across the Arctic Ocean is providing unprecedented opportunities to explore one of the least known ecosystems on Earth. In this Community Page, members of the EcoOmics project explain how data from MOSAiC will be used to study unique species, from viruses to multicellular animals.

## Teaser

Multiomics approaches need to be applied in the central Arctic Ocean to benchmark biodiversity change and to identify novel species and their genes. As part of MOSAiC, EcoOmics will therefore be essential for conservation and sustainable bioprospecting in one of the least explored ecosystems on Earth.

## The central Arctic Ocean and sampling for EcoOmics

Arctic ecosystems are among the most impacted on Earth by global warming. Air temperatures in the Arctic are rising 4 times faster than elsewhere on the globe [1]. The extent, thickness, and age of sea ice are all decreasing such that a considerable part of the Eurasian Arctic below 85°N is now ice free in summer, the remaining sea ice cover in the Arctic being thinner now than 2 decades ago [2]. Thus, the Arctic Ocean serves as a bellwether for the consequences of global change as well as the persistence of biodiversity on our planet. Yet, due to logistical and accessibility challenges, the Arctic—especially the central Arctic Ocean—remains one of the most poorly understood environments on Earth.

Moreover, accelerating environmental change in the Arctic has focused geopolitical interests due to increased access to biological and geological resources. The Arctic also provides significant ecosystem services based in part on its biodiversity, which is largely unknown [3,4]. In particular, marine microbes in sea ice and seawater are a cornerstone of this ecosystem and have pivotal roles in climate feedbacks and in sustaining food webs, which are central for conservation and ecosystem services. Microbes also serve as biological indicators due to their fast adaptive response to environmental change.

**Fig 1:**
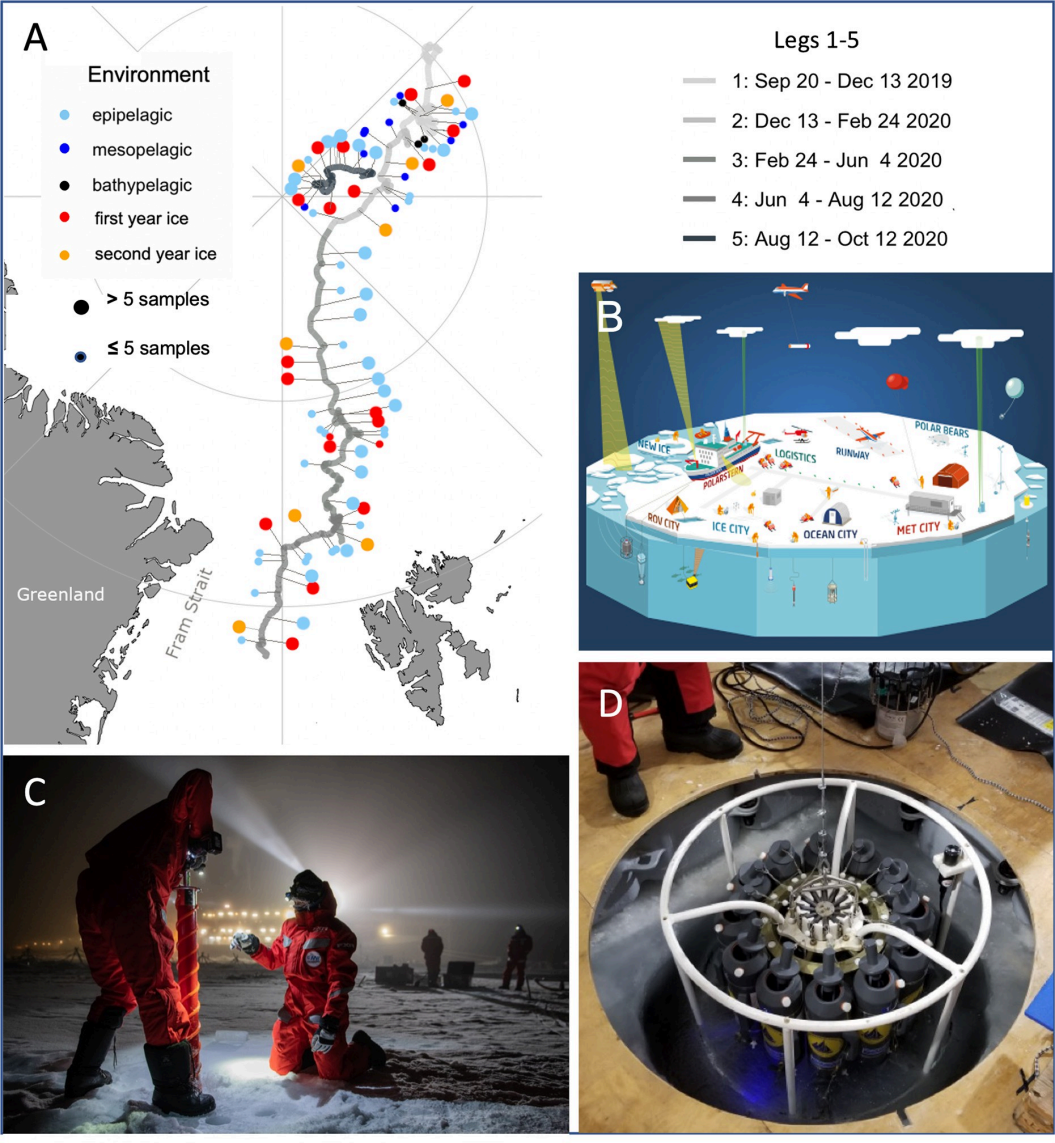
The MOSAiC expedition. (A) Map showing the route of the Polarstern during its drift throughout the MOSAiC expedition, with pins indicating sampling sites. Daily surface water sampling is shown aggregated by week. Maps were created with ggOceanMaps using basemaps from Natural Earth [8]. (B) Infographic of the drifting ice camp representing diverse scientific groups and their “cities” on the surface of sea ice. Credits Alfred-Wegener Institute. (C) Drilling of sea-ice cores in the dark during leg 2 (December 15, 2019). Credits Esther Horvath. (D) CTD (conductivity, temperature, and depth) rosette for measuring and sampling the sea water underneath sea ice as part of Ocean City. The CTD rosette is lowered through a hole (ca. 1.4 m in diameter) in the sea ice. Credits Ying-Chih Fang.

Multiomics approaches offer breadth and analytical resolution to benchmark and monitor biodiversity change due to global warming. These tools are also necessary for conservation (e.g., identification of novel species and assessing their risk of extinction) to mitigate the loss of biodiversity, as well as for bioprospecting (e.g., novel enzymes, antibiotics) to face the shortage of antibiotics and antivirals. In an effort to address these challenges, we have assembled the EcoOmics group, an international team of researchers who are part of the MOSAiC (Multidisciplinary drifting Observatory for the Study of Arctic Climate) expedition (2019 to 2020) [5]. EcoOmics applies high-throughput and high-resolution multiomics to reveal a year in the biological life of the central Arctic Ocean (Fig 1) with emphasis on microbiomes (a community of microorganisms living together in a habitat). The EcoOmics sampling scheme focuses on a 12-month time series with weekly resolution, supplemented by punctuated sampling of “special events,” characterised by short-term environmental changes significantly contributing to local differences in the sea-ice environment surrounding the drifting platform RV *Polarstern* (Fig 1A and 1B). Examples include the formation of leads, ridges, melt ponds on the ice, and the build-up of freshwater lenses floating on the ocean surface. During MOSAiC, scientists faced a variety of scientific and operational challenges when sampling related to the pronounced seasonality in light, temperature, and transpolar drift forces (Fig 1). The multiomics data will be analysed in an ecosystem context, enabling genotype-to-phenotype links and gene-to-ecosystem modelling [6,7]. The latter is based on novel computational frameworks that can integrate biological data with environmental conditions and larger-scale ecosystem processes, such as seasonality and associated habitat transformations (e.g., freeze–thaw cycles) [7].

## Resources and tools for revealing the life of biological communities in the Arctic Ocean

**Fig 2:**
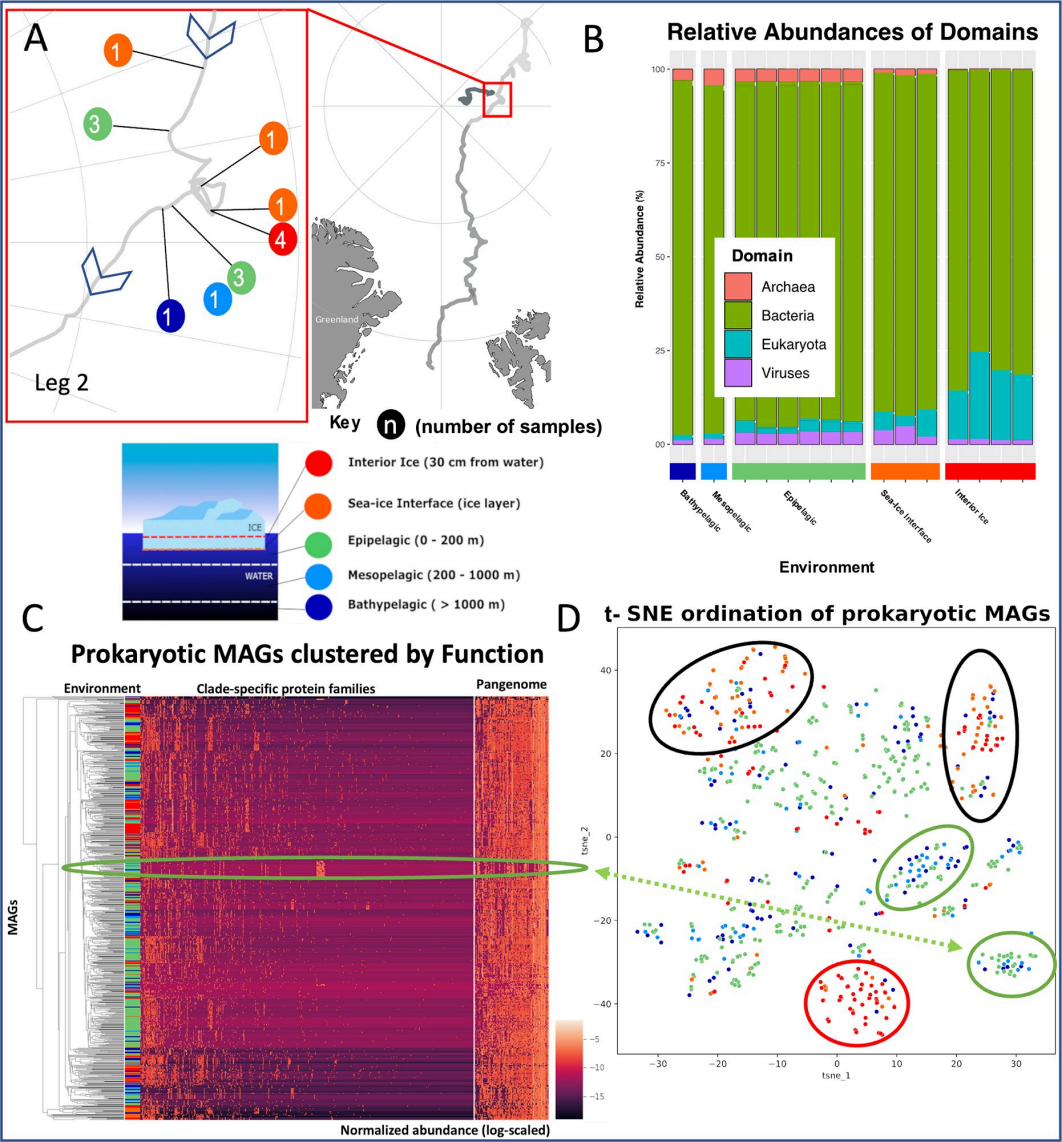
The EcoOmics project. (A) Detailed map of EcoOmics pilot sample sites, taken during Leg 2 of the expedition (December 13–February 24). Samples were collected from 5 different ice and water environments, with 2 epipelagic samples pooled from the other 4 samples. (B) Taxonomic abundances, based on the JGI IMG/M taxonomic annotation pipeline. Samples are dominated by bacteria, with a notable enrichment of eukaryotes in the interior sea ice. These same samples are noticeably lacking archaea. (C) Heatmap showing prokaryotic metagenome-assembled-genomes (MAGs) clustered based on abundances of protein families (Pfams). The highlighted group of protein families are present in significant proportions in a large clade of archaeal MAGs; this group contains a number of ribosome related Pfams specific to archaea. (D) t-SNE ordination to represent environmental filtering for prokaryotic MAGs, based on the abundances of their protein families. A large clade of archaeal MAGs forms a noticeable cluster (bottom right, circled). Some clades are found only in specific environments (green circle = pelagic environment; red circle = sea-ice environment), while others are found more widely such as alpha- and gamma-proteobacteria (black circle = present in all sampled environments). Data used in this manuscript was produced as part of the international Multidisciplinary drifting Observatory for the Study of the Arctic Climate (MOSAiC) with the tag MOSAiC20192020 and the Project_ID: AWI_PS122_00.

MOSAiC endorses data sharing within the consortium in addition to findable, accessible, interoperable and reusable (FAIR) principles of scientific data management [9]. Not only will the publication of scientific results take place under conditions of gold open access, MOSAiC EcoOmics will publish field and laboratory methods including their standard operating procedures, code, and data to provide a diverse set of resources to be used for future omics studies in polar oceans. Hence, we encourage diverse communities of researchers to make use of this unprecedented multiomics resource. As EcoOmics will benchmark sequence-led research in polar ecosystems (Fig 2), it will be instrumental to provide the resources and tools for future endeavours extending our initiative. Meta-barcoding, meta-genomic, and meta-transcriptomic sequencing for the seasonal time series, including basic bioinformatics and sequence analyses is a joint effort of Alfred-Wegener Institute (AWI, Germany), German Research Foundation (DFG), the Joint Genome Institute (JGI, USA), and numerous other instutions. Raw sequences generated by AWI and DFG will be published via GfBio, while related processed data will be published via PANGAEA. Sequencing information generated by JGI, including both raw sequencing output and analysis products, will be published and released according to the JGI data policy. Sequencing results will be integrated with a wealth of biological, ecological, chemical, and physical data generated by all research teams of MOSAiC.

Overall, the integration of information from meta-barcoding, meta-genomes, and meta-transcriptomes with well-coordinated environmental sampling will allow us to address the role of microbial and also metazoan biodiversity for Arctic marine ecosystem functioning and services at different levels and for various habitats over the complete seasonal cycle (Fig 1 and 2A). This integrative scientific approach is unprecedented for polar oceans. However, it is required to improve our projections of interacting species’ responses to climate change in the Arctic. Benchmarking these biotic responses requires us to catalogue as many of the microbial taxa in the central Arctic Ocean as possible. This is being addressed by a combination of barcoding sequencing based on phylogenetic marker genes (e.g., 16 and 18S rDNA), eDNA, and metagenome-assembled genomes (MAGs) (Fig 2) [4]. Preliminary results based on 18S-metabarcoding data and pilot metagenome sequencing have provided first evidence of habitat filtering in the Arctic Ocean likely caused by significantly different habitat characteristics (Fig 2B–2D) [10]. Thus, the latter shape microbiomes and likely also their activity, including feedbacks on the Arctic food web. Furthermore, functional insights based on the identification of protein families (Pfams) in prokaryotic MAGs revealed that the central Arctic Ocean is a treasure trove for discovering novel biology which likely has evolved because of adaptive processes required to thrive in this harsh environment (Fig 2C). Previous work corroborates the idea that life in polar oceans is characterised by unifying biological principles that are polar specific [11]. However, their discovery is still in its infancy. The integration of these principles with the highly variable physico-chemical nature of the central Arctic Ocean through space and time will allow us to identify what drives the variation in the number of species and traits across different ocean habitats in the central Arctic in a temporal context. Only then will we be able to predict the future of microbial taxa and their diverse populations with greater confidence in a warming Arctic Ocean.

## Conclusion

MOSAiC EcoOmics is well placed to build the most comprehensive and integrative genetic and genomic inventory of any polar ecosystem on Earth. Thus, EcoOmics will contribute to conservation efforts and extend fundamental questions in biology including the evolution of life on planet Earth, which remains incomplete unless polar organisms are considered. Those organisms are likely a treasure throve for discovering novel biology because of their unique adaptation. How our understanding of global biodiversity will be influenced by novel polar biology remains to be seen but our preliminary insights hold great promise.
